# Combined Use of Deep Eutectic Solvents, Macroporous Resins, and Preparative Liquid Chromatography for the Isolation and Purification of Flavonoids and 20-Hydroxyecdysone from *Chenopodium quinoa* Willd

**DOI:** 10.3390/biom9120776

**Published:** 2019-11-25

**Authors:** Jia Zeng, Xianchao Shang, Peng Zhang, Hongwei Wang, Yanlong Gu, Jia-Neng Tan

**Affiliations:** 1 Tobacco Research Institute, Chinese Academy of Agricultural Sciences, Qingdao 266101, China; zj253700112@126.com (J.Z.); sxc3220341@163.com (X.S.); Zhangpeng2008@caas.cn (P.Z.); 2 Graduate School of Chinese Academy of Agricultural Sciences, Beijing 100081, China; 3 State Key Laboratory Cultivation Base, Shandong Provincial Key Laboratory of Ophthalmology, Shandong Eye Institute, Shandong First Medical University & Shandong Academy of Medical Sciences, Qingdao 266071, China; 4 Key Laboratory of Energy Conversion and Storage, Ministry of Education, School of Chemistry and Chemical Engineering, Huazhong University of Science and Technology, Wuhan 430074, China

**Keywords:** DESs, preparative-scale purification, flavonoids, 20-hydroxyecdysone, *Chenopodium quinoa*

## Abstract

Deep eutectic solvents (DESs) were used in combination with macroporous resins to isolate and purify flavonoids and 20-hydroxyecdysone from *Chenopodium quinoa* Willd by preparative high-performance liquid chromatography (HPLC). The extraction performances of six DESs and the adsorption/desorption performances of five resins (AB-8, D101, HPD 400, HPD 600, and NKA-9) were investigated using the total flavonoid and 20-hydroxyecdysone extraction yields as the evaluation criteria, and the best-performing DES (choline chloride/urea, DES-6) and macroporous resin (D101) were further employed for phytochemical extraction and DES removal, respectively. The purified extract was subjected to preparative HPLC, and the five collected fractions were purified in a successive round of preparative HPLC to isolate three flavonoids and 20-hydroxyecdysone, which were identified by spectroscopic techniques. The use of a DES in this study significantly facilitated the preparative-scale isolation and purification of polar phytochemicals from complex plant systems.

## 1. Introduction

Deep eutectic solvents (DESs) exhibit the advantages of negligible volatility, adjustable viscosity, high solubility, preparation simplicity, eco-friendliness, and biodegradability, and have therefore attracted much attention [1,2,3,4,5,6]. Typically, DESs are homogeneous solutions of quaternary ammonium salts, which act as hydrogen-bond acceptors (HBAs) and engage in strong intermolecular hydrogen-bonding interactions with different hydrogen-bond donors. Among the diverse HBAs, cheap, biodegradable, and non-toxic choline chloride (ChCl) is most commonly used, [7] and ChCl-based DESs have been widely applied as reaction media in the fields of organic synthesis, biomass refinery, polymerization, and materials science [8,9,10,11,12,13]. Moreover, DESs can be used to extract phytochemicals, such as flavonoids, phenolic acids, κ-carrageenan, anthocyanins, and saponins, from various types of natural sources [14,15,16,17,18,19]. DES extraction technologies, however, have all been restricted to the analysis of active constituents, which could only be detected by HPLC, HPLC-MS/MS, or some other analysis instruments using standard chemicals. Until now, there have been almost no studies on the preparative-scale separation and purification of monomer compounds that are not available on-hand, particularly for plant samples, based on DES extraction. Thus, introducing DESs into the area of phytochemistry will provide an interesting approach for researchers.

*Chenopodium quinoa* Willd. (quinoa) is one of the oldest cultivated plants in the Andean region and is well known as a functional food and nutraceutical source rich in essential amino acids, unsaturated fats, phytoecdysteroids, flavonoids, phenolic acids, betalains, and saponins, which can allegedly reduce the risk of cardiovascular disease, cancer, osteoporosis, and diabetes [20,21,22,23,24,25,26,27,28,29,30,31]. For example, flavonoids are commonly used to treat and prevent diabetes and obesity [32,33], while 20-hydroxyecdysone is believed to exhibit antioxidant, antidiabetic, anti-obesity, antihypertensive, anticancer, and anti-inflammatory properties [34,35]. Therefore, the isolation and characterization of phytochemicals from *C. quinoa* is of high practical significance.

The extraction of phytochemicals from *C. quinoa* has gained significant attention [28,29,30]. For example, Dini et al. isolated and characterized phenolic constituents (e.g., kaempferol, quercetin (QE), and vanillic acid glycosides) from quinoa seeds [29] by partitioning the methanolic seed extract between *n*-butanol and water. The *n*-butanol phase was subjected to defatting with chloroform (CHC1_3_), and the obtained residue was loaded onto a Sephadex LH-20 column and subjected to droplet counter-current chromatography assisted by thin-layer chromatography to isolate several individual glycosides. In a report by Ho et al., where a series of ecdysteroids were isolated from *C. quinoa* [30], a suspension of the ethanolic (95 vol% aqueous ethanol) extract of ground seeds in water was sequentially treated with hexane, ethyl acetate, and *n*-butanol. Ecdysteroids, including 20-hydroxyecdysone, were separated from the *n*-butanol fraction by the combined use of Diaion HP-20 gel, silica gel, and RP-18 reverse-phase column chromatography. Notably, the above-mentioned organic solvent-based purification procedures are not efficient due to low extraction efficiencies and long extraction times. Therefore, the development of more efficient alternatives for the practical extraction of phytochemicals from *C. quinoa* is required.

**Figure 1:**
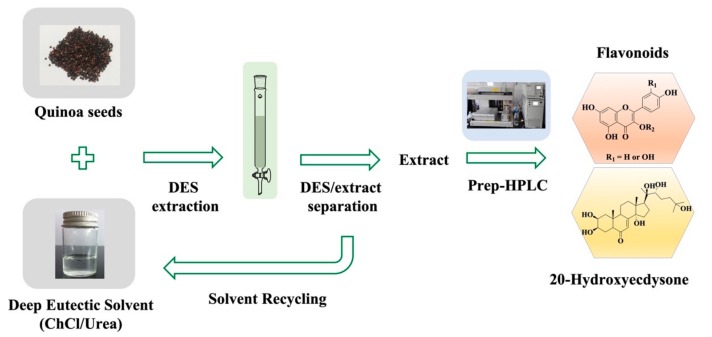
Schematic for routine isolation and purification of flavonoids and 20-hydroxyecdysone using the developed technique.

Herein, we achieved the separation and purification of flavonoids and 20-hydroxyecdysone from *C. quinoa* by using DESs as extracting solvents in conjunction with the use of macroporous resins, employing preparative high-performance liquid chromatography (HPLC) (Figure 1). The extraction performances of six DESs were investigated and compared with some selected organic solvents. The best DES was thereafter employed in some downstream experiments. A mixture consisting of the DES and quinoa extract was separated using a macroporous resin. Then, the extract was subjected to preparative HPLC for the isolation and purification of monomer compounds, which were further identified by spectroscopic techniques based on comparison with previously reported data.

## 2. Materials and Methods

### 2.1. Chemicals, Reagents, and Instruments

ChCl, aminoethyl alcohol, D-glucose, glycerol, oxalic acid, lactic acid, urea, methanol-*d*_4_, QE, 20-hydroxyecdysone, sodium nitrite, and aluminium trichloride (AlCl_3_) were purchased from Aladdin Industrial Co., Ltd. (Shanghai, China). HPLC-grade methanol and acetonitrile (ACN) were purchased from Shanghai Macklin Biochemical Co., Ltd. (Shanghai, China). Macroporous resins (D101, HPD 600, NKA-9, HPD 400, and AB-8) and analytical-grade methanol were purchased from Sinopharm Chemical Reagent Co., Ltd. (Shanghai, China). Deionized water was obtained using a Unique-R20 purification system (Xiamen RSJ Scientific Instruments Co., Ltd., Xiamen, China).

Nuclear magnetic resonance (NMR) spectra were recorded on an Agilent DD2 spectrometer (500 MHz for ^1^H and 125 MHz for ^13^C NMR; Agilent, CA, USA). High-resolution electrospray ionization–tandem mass spectrometry (HRESI-MS) was performed using an LTQ Orbitrap XL spectrometer (Thermo Scientific, MA, USA).

### 2.2. Determination of Total Flavonoid (TF) and 20-Hydroxyecdysone Contents

TF content was determined using a method obtained by the modification of a previously reported method [36]. A 96-well plate was sequentially charged with the concentrate (50 μL), 0.066 M sodium nitrite solution (100 μL), and 10% AlCl_3_ solution (*w*/*v*, 15 μL) and incubated at room temperature for 6 min. Thereafter, 0.5 M aqueous sodium hydroxide (NaOH, 100 μL) was added to terminate the reaction, and the absorbance of the reaction mixture at 510 nm was measured by a microplate reader using QE as a standard. TF content was expressed as milligrams of QE per gram of dry quinoa material.

20-Hydroxyecdysone content was quantified by ultra-performance liquid chromatography–triple quadrupole tandem mass spectrometry (UPLC-QqQ-MS/MS) using an Acquity UPLC system (Waters Corp., MA, USA) coupled with a TSQ Quantum triple quadrupole tandem mass spectrometer (Thermo Scientific, CA, USA). The mobile phase, comprising ACN (eluent A) and 0.1 vol% aqueous formic acid (eluent B), was supplied at a flow rate of 0.3 mL/min. The following gradient program was used: 0–2 min, 5–10 vol% ACN; 2–4 min, 10–20 vol% ACN; 4–6 min, 20–30 vol% ACN; 6–8 min, 30 vol% ACN; 8–10 min, 30–5 vol% ACN. The injection volume and column temperature were 10 µL and 25 °C, respectively.

Low-resolution mass spectrometric detection was carried out in the selected reaction-monitoring mode using an electrospray ionization (ESI) interface. The optimized parameters corresponded to a vaporizer temperature of 450 °C, corona discharge voltage of 4.0 kV, capillary temperature of 225 °C, sheath nitrogen gas pressure of 17 psi, auxiliary nitrogen gas pressure of 5 psi, and collision gas pressure of 1.5 mTorr. The Xcalibur software was used to control the LC-MS system and analyze the collected data. The mass spectrometric analysis of 20-hydroxyecdysone was initially performed over the full scan range in a negative-ion mode, and a pseudomolecular ion at *m/z* 479.176 (*t*_R_ = 4.65 min) as well as four product fragment ions at *m/z* 319.1 (collision energy = 27 eV), 301.1 (collision energy = 33 eV), 159.0 (collision energy = 27 eV), and 83.0 (collision energy = 37 eV) were detected. The fragmentation patterns of 20-hydroxyecdysone were compared with those previously reported in the literature [37].

### 2.3. Preparation and Selection of DESs

The ChCl/urea DES was prepared using a modification of a previously reported method [12] (Table 1). Briefly, a glass vial was charged with ChCl and urea, and the obtained mixture was stirred at 280 rpm at 80 °C for 2 h. The obtained ChCl/urea DES (clear stable liquid) was mixed with water (7/3, *v*/*v*), and the solution was used for further experiments. Other DESs were prepared in a similar way.

Ultrasound-assisted extraction was used to extract TF and 20-hydroxyecdysone. The extraction capacities of six DESs were evaluated using the extraction yields (*E*_y_) of TF and 20-hydroxyecdysone. First, 100 mg of quinoa seed powder and 1.0 mL of DES were added to a 10 mL glass tube in sequence. The mixture was placed on a vortex meter, stirred for 2 min, and further ultrasonicated at 50 °C for 30 min (25 kHz, 200 W). After the tube cooled down to the room temperature, the mixture was centrifuged at 3000 rpm for 10 min. The supernatant (1 mL) was diluted with methanol (4 mL), and the mixture was passed through a 0.22 μm filter for subsequent analyses. Each extraction was performed in triplicate, and E_y_ was calculated as follows:

*E*_*y*_ = (*C*_0_ × *V*_0_)/*M*_0_, (1)

where *C*_0_ is the concentration of TF or 20-hydroxyecdysone found in the DES, *V*_0_ is the volume of the diluted liquid, and *M*_0_ is the mass of the sample.

### 2.4. Response Surface Methodology (RSM)

The optimal values for three variables in the selected DES, including water content (*a*), extraction temperature (*b*), and solid (quinoa seed)–liquid (DES) ratio (*c*) at three levels (–1, 0, and +1) were determined using RSM based on a Box–Behnken design (BBD). Table S1 (Supplementary Materials) shows the investigated variables and their values in the three-level BBD. The experiment was designed to reveal the effect of each parameter on TF and 20-hydroxyecdysone contents as well as the interactions between the three main factors. The entire study was comprised of 17 separate experiments including 5 center points, each of which was conducted in triplicate.

Statistical comparisons were made using a single factor analysis of variance (ANOVA); *p*-values < 0.05 were considered significant. Data were processed using Design-Expert 8.5 statistical software and the social package for statistical studies (SPSS 19.0).

### 2.5. Adsorption/Desorption Capacities of the Five Employed Resins

The adsorption/desorption capacities of the five kinds of macroporous resins were investigated using the adsorption/desorption yields of TF and 20-hydroxyecdysone. The DES extract solution was added to a 200 mL flask containing 1.0 g of the pretreated resins. The mixture was shaken at 120 rpm at room temperature for 24 h to reach adsorption equilibrium, and the resins were washed by deionized water and then desorbed with 50 mL of methanol in the flask, which was continually shaken at 120 rpm at room temperature for 24 h. The adsorption/desorption yields of resins were calculated according to the following Equations (2) and (3):

*D*_a_ = (*C*_0_ − *C*_a_)*/C*_0_, (2)

*D*_d_ = *C*_d_ × *V*_d_*/*(*C*_0_ − *C*_a_) × *V*_0_, (3)

where *D*_a_ is the adsorption yield at adsorption equilibrium; *D*_d_ is the desorption yield after adsorption equilibrium; *C*_0_, *C*_a_, and *C*_d_ represent the content of TF or 20-hydroxyecdysone at initial equilibrium, absorption equilibrium, and desorption equilibrium, respectively; *V*_0_ and *V*_d_ are the initial sample volume and desorption solution volume (mL), respectively.

### 2.6. Small- and Preparative-Scale Sample Extraction

Quinoa seeds, desaponified by soaking in distilled water for 24 h without the presence of foam, were purchased from Xinjing Quinoa Cultivation and Promotion Co., Ltd. (Shanxi, China) [38]. For small-scale extraction, the seeds were ground and 1.0 g of the obtained powder was mixed with aqueous DES (10 mL). The resulting mixture was sonicated (100 W) at 50 °C for 30 min in an ultrasonic bath (Scientz SB25-12D, Ningbo Scientz Biotechnology Co., Ltd, Ningbo, China) and then centrifuged at 5000 rpm for 10 min. Small-scale extraction was used in the selection of DESs and macroporous resins as well as the RSM experiments. For preparative-scale extraction, powdered seeds (1.0 kg) were suspended in 10 L of aqueous DES. The extraction process was similar to that of small-scale extraction. The collected supernatants were loaded onto a macroporous resin column (60 mm × 1200 mm), which was sequentially flushed with deionized water (5000 mL) at a flow rate of 15 mL/min (to remove the DES) and methanol (2000 mL) at a flow rate of 20 mL/min under reduced pressure. The methanolic extract was concentrated, and the concentrate was stored at 4 °C for further use.

### 2.7. Analytical and Preparative HPLC

For the first separation, analytical HPLC of quinoa extract was performed on a Waters 2489 chromatography system (Waters, MA, USA) equipped with a C18 separation column (5 µm, 4.6 × 150 mm) using ACN and water for gradient elution at a flow rate of 1.0 mL/min. UV detection was performed at 210 nm. The gradient program was as follows: 0–5 min, 8 vol% ACN; 5–7.5 min, 8–15 vol% ACN; 7.5–10 min, 15–30 vol% ACN; 10–15 min, 30–50 vol% ACN; 15–17.5 min, 50–60 vol% ACN; 17.5–30 min, 60 vol% ACN. The first-round preparative HPLC of quinoa extract was performed on a DAC100 preparative HPLC system (Beijing, China) equipped with a C18 separation column (5 µm, 100 × 250 mm) at a flow rate of 300 mL/min, while the other chromatographic conditions were identical to those used for analytical HPLC. The obtained eluent was separated into five (I–V) fractions.

In the second round of separation, fractions I–V were subjected to analytical and preparative HPLC performed on the Waters 2489 chromatography system equipped with 4.6 × 150 mm (5 µm) and 10 × 250 mm (5 µm) C18 columns, respectively. The flow rates of analytical and preparative separations were set to 1.0 and 6.0 mL/min, respectively. The mobile phase composition and UV detection parameters were identical to those used for the first round of separation. The gradient program was as follows: 0–5 min, 5 vol% ACN; 5–10 min, 5–10 vol% ACN; 10–15 min, 10 vol% ACN; 15–20 min, 10–15 vol% ACN; 20–25 min, 15 vol% ACN; 25–30 min, 15–30 vol% ACN; 30–35 min, 30 vol% ACN; 35–40 min, 30–50 vol% ACN; 40–50 min, 50 vol% ACN.

## 3. Results and Discussion

### 3.1. Preparation and Optimization of DES Systems

The non-toxicity, biodegradability, and low cost of ChCl make it suitable for combination with a series of hydrogen-bond donors (HBDs) to afford biocompatible and renewable DESs. Herein, six DESs were synthesized by combining ChCl with HBDs belonging to the classes of inexpensive and easily available fine chemicals (aminoethyl alcohol and urea), polyalcohols (glycerol), organic acids (oxalic and lactic acids), and sugars (D-glucose). The composition and physicochemical properties (density, conductivity, and viscosity) of the six DESs are listed in Table 1.

**Figure 2:**
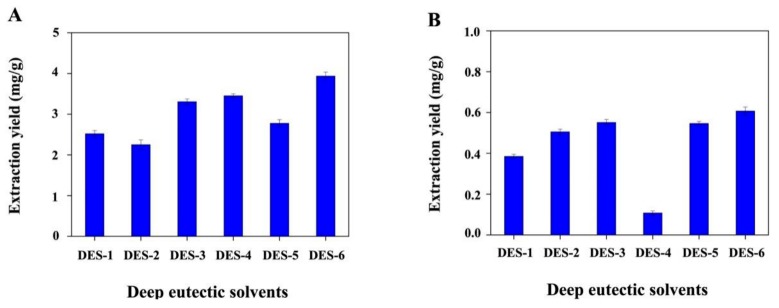
(**A**) Total flavonoid (TF) and (**B**) 20-hydroxyecdysone extraction efficiencies of the employed DESs. TF extraction efficiency was expressed as milligrams QE equivalents per gram of quinoa sample.

DES extraction performance was evaluated by considering the TF and 20-hydroxyecdysone contents of the corresponding extracts. Figure 2A compares the obtained TF contents, revealing that the value obtained for DES-6 (3.93 ± 0.15 mg/g) exceeded those obtained for DES-4 (3.45 ± 0.09 mg/g), DES-3 (3.31 ± 0.10 mg quercetin equivalents (QE)/g), DES-5 (2.78 ± 0.17 mg QE/g), DES-1 (2.52 ± 0.06 mg QE/g), and DES-2 (2.25 ± 0.13 mg QE/g). Dini et al. [39] determined the TF contents before and after cooking in sweet and bitter quinoa seeds. The samples were extracted in methanol–water (80:20 *v*/*v*). It was found that the bitter quinoa seeds processed higher TF content before cooking (1.39 ± 0.35 mg catechin equivalents (CE)/1.0 g) than the sweet seeds (0.81 ± 0.10 mg CE/1.0 g). After cooking, TF contents of bitter and sweet quinoa seeds decreased significantly to 0.63 ± 0.15 mg CE/g and 0.18 ± 0.07 mg CE/g, respectively. In consideration of the similar molecular weights of QE and CE, the TF contents obtained by Dini et al. were lower than those in our study. Figure 2B shows that similar 20-hydroxyecdysone contents were obtained for DES-3 (551.50 ± 4.51 mg/g), DES-5 (546.33 ± 7.60 mg/g), and DES-2 (504.69 ± 12.05 mg/g), while higher values were obtained for DES-6 (606.76 ± 7.98 mg/g), and significantly lower values were obtained for DES-1 (384.80 ± 12.16 mg/g) and DES-4 (107.65 ± 2.88 mg/g). Lafont et al. [30] determined the 20-hydroxyecdysone contents of quinoa seeds obtained from four producing countries (Bolivia, Chile, Ecuador, and Peru). Seed powder was extracted with 20 mL methanol–water (35:65, *v*/*v*) overnight with magnetic stirring. The obtained 20-hydroxyecdysone contents (0.32–0.42 mg/g) were significantly lower than those obtained after extraction by DES-2, DES-3, DES-5, and DES-6. In addition, the 20-hydroxyecdysone content of the DES-6 extract was also much higher than the values previously reported by Raskin et al. (0.18–0.49 mg/g) [28]. It is possible that the weakly alkaline HBD (urea) possessed stronger hydrogen-bond interactions with phenolic hydroxyls and alcoholic hydroxyls in the flavonoids or alcoholic hydroxyls in 20-hydroxyecdysone than the neutral (D-glucose and glycerol), strong alkaline (aminoethyl alcohol), and weakly acidic (oxalic acid and lactic acid) HBDs, contributing to the enhanced extraction yields. Based on the above results, DES-6 (ChCl/urea) was used in subsequent experiments.

### 3.2. BBD Experimental Design

Three values, including water content in the DES (variable *a*), extraction temperature (variable *b*), and solid–liquid ratio (variable *c*), were used as independent variables to efficiently optimize the extraction yields of TF and 20-hydroxyecdysone from *C. quinoa* in the RSM experiments. The extraction yields were considered as related responses in order to evaluate the efficiency of the extraction procedure. The experiments were performed in a random order to avoid systematic error. The results, including the coded variables and related responses, are presented in Table 2. A second-order polynomial equation was applied to express the proposed model after multiple regression analysis of the experimental data. The regression model equations for the responses and variables in terms of the coded levels are as follows:

*Y*_TF_ = 3.94 + 0.48*a* + 0.017*b* − 0.25*c*-0.097*ab* − 0.028*ac* + 0.20*bc* − 0.86*a*^2^ − 0.41*b*^2^ − 0.34*c*^2^, (4)

*Y*_20-Hydroxyecdysone_ = 0.50 + 0.033*a* − 0.017*b* − 0.025*c* − 0.037*ab* − 0.020*ac* − 0.046*bc* -0.068*a*^2^ − 0.026*b*^2^ − 0.12*c*^2^, (5)

where *a* is the DES water content, *b* is the extraction temperature, and *c* is the solid–liquid ratio.

Variances of the ANOVA regression model equations of TF and 20-hydroxyecdysone are shown in Tables S2 and S3 (Supplementary Materials). The coefficients (*R*^2^) of the variables of response were 0.9553 and 0.9501 for TF and 20-hydroxyecdysone, respectively. The *F*-values for the lack-of-fit model were all non-significant, which supported our assumption that the models were sufficient to accurately represent the experimental data.

**Figure 3:**
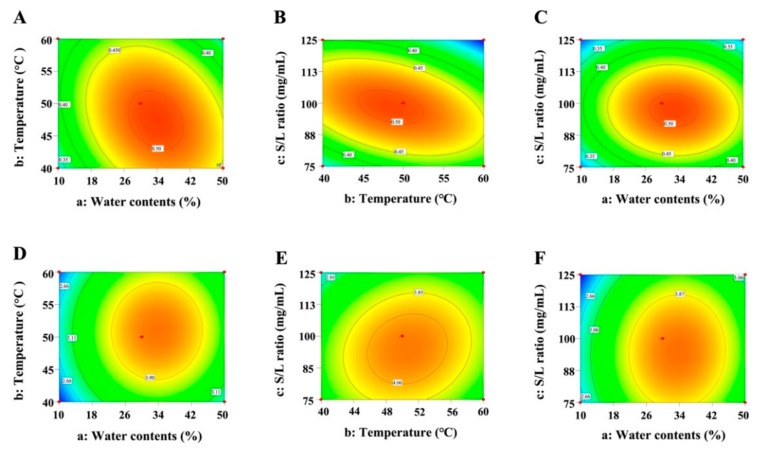
Response surface plots of the models for (**A**, **B**, and **C**) TF and (**D**, **E**, and **F**) 20-hydroxyecdysone contents.

A response surface plot of the model was used for graphically interpreting the significant effects of interactions among the three variables on the contents of TF and 20-hydroxyecdysone (Figure 3). Results showed that the extraction yields of TF and 20-hydroxyecdysone were apparently related to the main variable. In the model, the concentration of water (%) in the DES solution (variable *a*) and solid–liquid ratio (variable *c*) exhibited statistically significant effects (*p* < 0.05) on the extraction yields of TF and 20-hydroxyecdysone. In contrast, extraction temperature (variable *b*) showed a non-significant effect (*p* > 0.05) on the contents.

Based on RSM results, TF was extracted from *C. quinoa* using the optimum conditions (DES water content, 32.4%; extraction temperature, 50.7 °C; and solid–liquid ratio, 97.2 mg/mL). The optimal extraction conditions for 20-hydroxyecdysone extraction from *C. quinoa*, as well as the related maximal response values (Table 3), were established by applying a simple procedure (DES water content, 32.6%; extraction temperature, 47.8 °C; and solid–liquid ratio, 99.7 mg/mL). A verification experiment performed using these conditions gave optimal extraction yields of 4.23 and 0.51 mg/g for TF and 20-hydroxyecdysone, respectively. In order to simplify the operation, the experimental conditions with a DES water content of 30.0%, an extraction temperature of 50.0 °C, and a solid–liquid ratio of 100.0 mg/mL were selected for DES extraction (10 L). Under this condition, the extraction yields of TF and 20-hydroxyecdysone were 4.11 and 0.50 mg/g, respectively, which were close to the predicted values.

### 3.3. Macroporous Resin Selection

**Figure 4:**
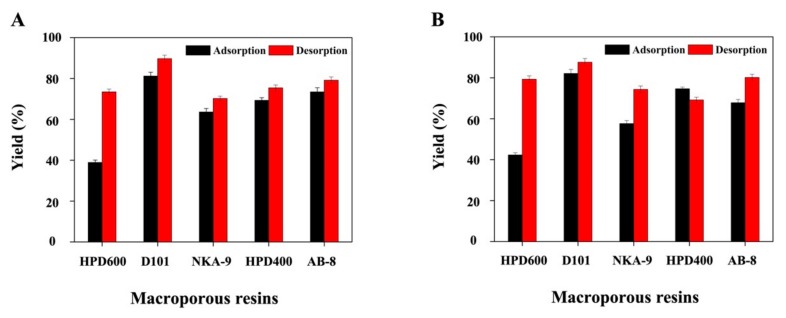
(**A**) TF and (**B**) 20-hydroxyecdysone adsorption/desorption capacities of the five employed resins.

Macroporous resins exhibit the advantages of easy recyclability, low cost, high adsorption/desorption capacity, and suitability for large-scale production, and have therefore been widely used for the enrichment and purification of phytochemicals extracted from complex plant systems. Here, we used TF and 20-hydroxyecdysone contents to evaluate the performances of five macroporous resins (AB-8, D101, HPD 400, HPD 600, and NKA-9) for removing DESs from extracts prior to preparative HPLC (Figure 4A,B). The D101 resin had the highest TF adsorption capacity (81.2%), which was approximately two-fold higher than that of the HPD 400 resin (38.9%) (Figure 4A). The adsorption capacities of AB-8 (73.4%) and HPD 400 (69.3%) were similar to each other and higher than that of NKA-9 (63.6%). The TF desorption capacities of all the tested resins exceeded 70%. In particular, D101 (89.7%) and AB-8 (79.2%) had similar desorption capacities that exceeded those of HPD 400 (75.4%), HPD 600 (73.4%), and NKA-9 (70.2%). Moreover, D101 exhibited the highest 20-hydroxyecdysone adsorption/desorption capacities among the five tested resins (Figure 4B). Therefore, D101 was selected for implementing the next studies.

### 3.4. Preparative-Scale Isolation and Purification of Monomer Compounds

**Figure 5:**
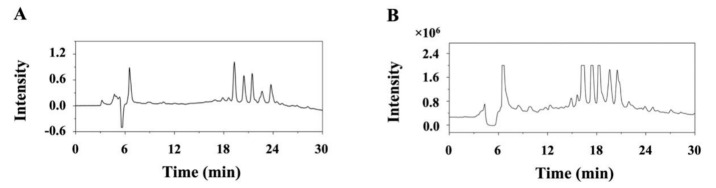
Analytical (**A**) and preparative-scale (**B**) HPLC-UV chromatograms of quinoa extracts.

**Figure 6:**
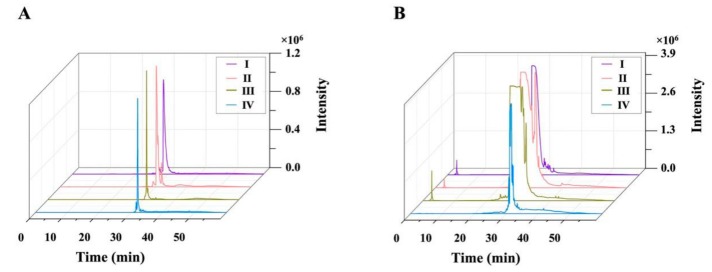
Analytical (**A**) and preparative-scale (**B**) HPLC-UV chromatograms of fractions I–IV.

Eluate concentrates obtained at the DES removal stage were sequentially subjected to analytical and preparative HPLC, and the corresponding chromatograms (Figure 5) showed that the resolution of preparative HPLC was similar to that of analytical HPLC. Fractions I (242 mg), II (253 mg), III (206 mg), IV (988 mg), and V (265 mg) separated from 9.2 g of the concentrate were further evaluated by analytical HPLC. The corresponding chromatograms, shown in Figure 6A, revealed that the compounds obtained after the first separation round were not sufficiently pure. Therefore, a second round of preparative HPLC (Figure 6B) was performed using a C18 column with a smaller inner diameter (5 µm, 10 × 250 mm), yielding compound **1** (48.7 mg), compound **2** (47.9 mg), compound **3** (39.9 mg), and compound **4** (230.6 mg) from fractions I–IV, respectively. No compound was isolated from fraction V.

### 3.5. Identification of Isolated Compounds

Compounds **1**–**4** were identified based on their HRESI-MS, ^1^H NMR, ^13^C NMR, and 2D NMR spectra (Table 4).

Compound **1**. HRESI-MS: found *m/z* 755.2048 [M-H]^−^, calcd. for C_33_H_40_O_20_ *m/z* 756.2113. ^1^H NMR (500 MHz, CD_3_OD): *δ* (ppm) 7.69 (1H, d, *J* = 2.2 Hz), 7.56 (1H, dd, *J* = 8.5, 2.2 Hz), 6.87 (1H, d, *J* = 8.5 Hz), 6.36 (1H, d, *J* = 2.1 Hz), 6.19 (1H, d, *J* = 2.2 Hz), 5.66 (1H, d, *J* = 7.8 Hz), 5.59 (1H, d, *J* = 7.7 Hz), 5.21 (1H, d, *J* = 1.4 Hz), 4.54 (1H, d, *J* = 1.5 Hz), 1.18 (3H, d, *J* = 6.5 Hz), and 0.95 (3H, d, *J* = 6.5 Hz). ^13^C NMR (125 MHz, CD_3_OD): *δ* (ppm) 178.1, 164.3, 161.7, 156.9, 156.9, 148.2, 144.4, 133.1, 121.9, 121.6, 115.9, 114.7, 104.5, 100.4, 99.6, 98.3, 98.3, 93.2, 76.0, 74.2, 73.8, 72.6, 72.5, 71.0, 70.9, 70.8, 70.6, 69.4, 68.4, 68.3, 65.3, 16.6, 16.0. A comparison with previously reported data [29] allowed compound **1** to be identified as quercetin-3-*O*-(2,6-di-α-L-rhamnopyranosyl)-β-D-galactopyranoside (Figures S4–S6).

Compound **2**. HRESI-MS: found *m/z* 741.1875 [M-H]^−^, calcd. for C_32_H_38_O_20_ *m/z* 742.1956. ^1^H NMR (500 MHz, CD_3_OD): *δ* (ppm) 7.71 (1H, d, *J* = 2.1 Hz), 7.62 (1H, d, *J* = 8.4, 2.1 Hz), 6.86 (1H, d, *J* = 8.4 Hz), 6.37 (1H, d, *J* = 1.9 Hz), 6.17 (1H, d, *J* = 1.9 Hz), 5.40 (1H, d, *J* = 7.9 Hz), 4.52 (1H, d, *J* = 1.5 Hz), 5.46 (1H, d, *J* = 1.2 Hz), 4.06 (1H, d, *J* = 1.2 Hz), 3.99 (1H, dd, *J* = 7.9, 9.4 Hz), 3.79 (1H, m), 3.78 (1H, d, *J* = 3.4 Hz), 3.71 (1H, m), 3.70 (1H, dd, *J* = 12.6, 4.7 Hz), 3.61 (2H, s), 3.57 (1H, dd, *J* = 3.4, 1.5 Hz), 3.55 (1H, d, *J* = 3.5 Hz), 3.51 (1H, dq, *J* = 9.4, 6.2 Hz), 3.42 (1H, dd, *J* = 12.6, 6.8 Hz), 3.27 (1H, t, *J* = 9.4 Hz), 1.18 (3H, d, *J* = 6.2 Hz). ^13^C NMR (125 MHz, CD_3_OD): *δ* (ppm) 178.0, 164.3, 161.3, 157.0, 156.9, 148.3, 144.4, 133.5, 121.8,121.8, 115.9, 114.7, 109.5, 104.3, 100.6, 100.3, 98.3, 93.2, 79.4, 76.6, 74.6, 74.2, 73.4, 73.3, 72.6, 71.8, 70.8, 69.3, 68.3, 65.5, 64.8, 16.5. The ^1^H and ^13^C NMR spectra of compound **2** were similar to those of quercetin-3-*O*-β-D-apiofuranosyl-(1‴→2″)-*O*-[α-L-rhamnopyranosyl-(1″″→6″)]-β-D-galactopyranoside-3′,4′-dimethyl ether reported by Dini et al. [29] and compound **2** was concluded to be the demethoxylated version of the above species, namely quercetin-3-*O*-β-D-apiofuranosyl-(1‴→2″)-*O*-[α-L-rhamnopyranosyl-(1″″→6″)]-β-D-galactopyranoside. As the NMR data of compound **2** have not been frequently reported, this compound was fully characterized by ^1^H, ^13^C, heteronuclear singular quantum correlation (HSQC), heteronuclear multiple bond correlation (HMBC), and ^1^H-^1^H correlated spectroscopy (COSY) NMR techniques, and the obtained results are shown in Table S4 (Figures S7–S14).

Compound **3**. HRESI-MS: found *m/z* 739.2099 [M-H]^−^, calcd. for C_33_H_40_O_19_ *m/z* 740.2164. ^1^H NMR (500 MHz, CD_3_OD): *δ* (ppm) 8.06 (2H, d, *J* = 8.9 Hz), 6.89 (2H, d, *J* = 8.9 Hz), 6.37 (1H, d, *J* = 1.7 Hz), 6.17 (1H, d, *J* = 1.5 Hz), 5.60 (1H, d, *J* = 7.6 Hz), 5.21 (1 H, d, *J* = 1.5 Hz), 4.52 (1H, d, *J* = 1.5 Hz), 1.17 (3H, d, *J* = 6.5 Hz) and 0.98 (3H, d, *J* = 6.5 Hz). ^13^C NMR (125 MHz, CD_3_OD): *δ* (ppm) 178.1, 164.3, 161.7, 159.9, 157.2, 157.0, 133.0, 130.8, 130.8, 121.6, 114.8, 114.8, 104.6, 101.1, 100.4, 99.4, 98.4, 93.3, 76.1, 74.3, 73.8, 72.6, 72.5, 71.0, 70.9, 70.9, 70.7, 69.3, 68.4, 68.3, 65.7, 16.5, 16.1. Comparison with previously reported data [29] allowed compound **3** to be identified as kaempferol-3-*O*-(2,6-di-α-L- rhamnopyranosyl)-β-D-galactopyranoside (Figures S15–S17).

Compound **4**. HRESI-MS: found *m/z* 479.3021 [M-H]^−^, calcd. for C_27_H_44_O_7_ *m/z* 480.3087. ^1^H NMR (500 MHz, CD_3_OD): *δ* (ppm) 5.80 (1H, d, *J* = 2.8 Hz), 3.95 (1H, br s), 3.83 (1H, br s), 1.20 (3H, s), 1.19 (3H, s), 1.18 (3H, s), 0.96 (3H, s), and 0.89 (3H, s). ^13^C NMR (125 MHz, CD_3_OD): *δ* (ppm) 205.0, 166.6, 120.7, 83.8, 77.0, 76.5, 69.9, 67.3, 67.1, 50.4, 49.1, 41.0, 37.8, 35.9, 33.7, 31.4, 31.1, 30.4, 28.3, 27.5, 25.9, 23.0, 20.1, 20.1, 19.6, 16.6. Apart from the missing carbon signal at 47.0 ppm, which was believed to overlap with the solvent peak, the spectral data were in accordance with those reported for 20-hydroxyecdysone [30]. Thus, compound **4** was identified as 20-hydroxyecdysone (Figures S18–S20).

## 4. Conclusions

A technique combining DES-assisted extraction, macroporous resin-facilitated DES removal, and preparative HPLC was developed for the preparative-scale isolation and purification of flavonoids and 20-hydroxyecdysone from *C. quinoa* Willd. Specifically, three flavonoids and 20-hydroxyecdysone were successfully separated and purified, and their structures were identified by HRESI-MS, 1D NMR, and 2D NMR. No saponins were isolated, as the employed *C. quinoa* sample had been subjected to desaponification. DESs were concluded to be appropriate for the extraction of polar compounds, such as glycosides, or other OH-group-rich compounds. The developed method is expected to be well suited for the preparative-scale separation and purification of minor phytochemicals of interest from complex plant systems. Further work will focus on the application of our method to the purification of triterpenoid saponins.

## Figures and Tables

**Table 1:** Compositions and physicochemical properties of deep eutectic solvents (DESs) used in this study.

DESs	Composite of DES	Molar Ratio	Viscosity ^a^ Pa·s	Conductivity μS/cm	Density g/cm^3^
DES-1	ChCl/aminoethyl alcohol	1:6	0.037	3330	1.058
DES-2	ChCl/D-glucose	1:1	- ^c^	98	1.304
DES-3	ChCl/glycerol	1:2	0.227	1750	1.171
DES-4	ChCl/oxalic acid ^b^	1:1	0.152	1043	1.230
DES-5	ChCl/lactic acid	1:1	0.270	3670	1.133
DES-6	ChCl/urea	1:2	0.658	1880	1.184

^a^ Determined at 30 °C. ^b^ Dehydrate. ^c^ Not available.

**Table 2:** Response surface optimization experiments using DES-6 extraction of investigated variables.

No.	Experiment Design						Response	
	Coded Values			Variables			Extraction Yields (mg/g)	
	a	b	c	a (%)	b (°C)	c (mg/mL)	TF	20-Hydroxyecdysone
1	1	0	1	50	50	125	2.90	0.29
2	0	1	1	30	60	125	3.20	0.25
3	0	0	0	30	50	100	4.49	0.51
4	−1	−1	0	10	40	100	2.08	0.33
5	0	0	0	30	50	100	4.29	0.53
6	1	1	0	50	60	100	3.07	0.34
7	0	0	0	30	50	100	4.13	0.50
8	1	0	−1	50	50	75	3.20	0.34
9	0	−1	1	30	40	125	2.72	0.38
10	0	1	−1	30	60	75	3.27	0.42
11	1	−1	0	50	40	100	2.78	0.47
12	−1	0	−1	10	50	75	2.53	0.30
13	−1	1	0	10	60	100	2.25	0.34
14	0	0	0	30	50	100	4.28	0.51
15	0	0	0	30	50	100	3.70	0.47
16	0	−1	−1	30	40	75	3.40	0.32
17	−1	0	1	10	50	125	2.03	0.29

**Table 3:** Optimal conditions of the variables that maximize the response values using response surface methodology.

	Optimal Variable Conditions			Optimum (mg/g)
	a (%)	b (°C)	c (mg/mL)	
TF	32.4	50.7	97.2	4.23
20-Hydroxyecdysone	32.6	47.8	99.7	0.51

**Table 4:** Structures of the isolated compounds.

Numbers	Compound Names		R_1_	R_2_
1	Quercetin-3-*O*-(2,6-di-α-L-rhamnopyranosyl)-β-D-galactopyranoside	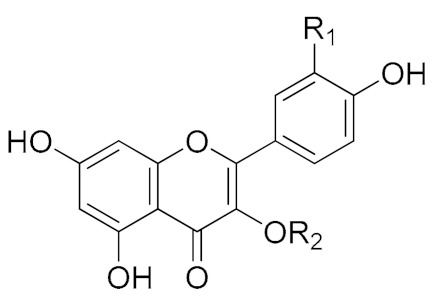	OH	3-*O*-(2,6-di-α-L-rhamnopyranosyl)-β-D-galactopyranoside
2	Quercetin-3-*O*-β-D-apiofuranosyl-(1‴→2")-*O*-[α-L-rhamnopyranosyl-(1″″→6″)]-β-D-galactopyranoside		OH	3-*O*-β-D-apiofuranosyl-(1‴→2″)-*O*-[α-L-rhamnopyranosyl-(1″″→6″)]-β-D-galactopyranoside
3	Kaempferol-3-*O*-(2,6-di-α-L- rhamnopyranosyl)-β-D-galactopyranoside		H	3-*O*-(2,6-di-α-L-rhamnopyranosyl)-β-D-galactopyranoside
4	20-Hydroxyecdysone	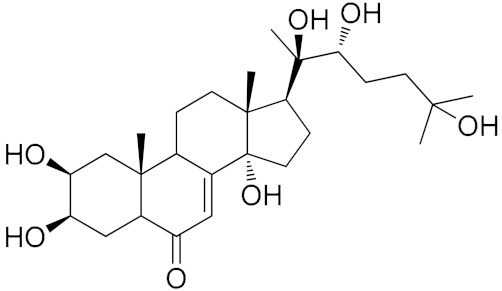		

## Supplementary Materials

The following are available online at https://www.mdpi.com/2218-273X/9/12/776/s1.
