# Supplementary material for: Combined Use of Deep Eutectic Solvents, Macroporous Resins, and Preparative Liquid Chromatography for the Isolation and Purification of Flavonoids and 20-Hydroxyecdysone from Chenopodium quinoa Willd
Source: Biomolecules. 2019 Nov 25;9(12):776. doi: 10.3390/biom9120776 (PMC6995548; doi:10.3390/biom9120776)
Supplement: Supplementary file 1 [file biomolecules-09-00776-s001.pdf]

SUMMARY

This supporting information file includes additional results and information as described in the text of the main article. Including:

Contents

Page S-3: Table S1. The investigated variables and their levels in three-level BBD.

Page S-4: Table S2. Analysis of variance for regression model equation (TF content).

Page S-5: Table S3. Analysis of variance for regression model equation (20-hydroxyecdysone content).

Page S-6: Table S4. NMR data of compound **2** in CD_3OD.

Page S-7: Figure S1. UPLC-QqQ-MS/MS spectrum of 20-hydroxecdysone.

Page S-8: Figure S2. Standard curve of 20-hydroxecdysone.

Page S-9: Figure S3. Standard curve of TF.

Page S-10: Figure S4. ^{1}H NMR of compound **1**.

Page S-11: Figure S5. ^{13}C NMR of compound **1**.

Page S-12: Figure S6. HRESI-MS of compound **1**.

Page S-13: Figure S7. ^{1}H NMR of compound **2**.

Page S-14: Figure S8. ^{13}C NMR of compound **2**.

Page S-15: Figure S9. DEPT (135°) of compound **2**.

Page S-16: Figure S10. DEPT (90°) of compound **2**.

Page S-17: Figure S11. ^{1}H-^{1}H COSY of compound **2**.

Page S-18: Figure S12. HMBC of compound **2**.

Page S-19: Figure S13. HSQC of compound **2**.

Page S-20: Figure S14. HRESI-MS of compound **2**.

Page S-21: Figure S15. ^{1}H NMR of compound **3**.

Page S-22: Figure S16. ^{13}C NMR of compound **3**.

Page S-23: Figure S17. HRESI-MS of compound **3**.

Page S-24: Figure S18. ^{1}H NMR of compound **4**.

Page S-25: Figure S19. ^{13}C NMR of compound **4**.

Page S-26: Figure S20. HRESI-MS of compound **4**.

Table S1. investigated variables and their levels in three-level BBD.

Variables	Levels		
	−1	0	1
Water content (%)	10	30	50
Temperature (°C)	40	50	60
Solid/liquid ratio (mg/mL)	75	100	125

Table S2. Analysis of variance for regression model equation (TF content).

Source	Sum of squares	df	Mean square	F value	*p*-value prob > F
Model	9.3115	9	1.0346	20.6183	0.0003
a	1.1640	1	1.1640	23.1966	0.0019
b	0.0827	1	0.0827	1.6489	0.2400
c	0.3030	1	0.3030	6.0378	0.0436
ab	0.0032	1	0.0032	0.0639	0.8077
ac	0.0090	1	0.0090	0.1795	0.6845
bc	0.0939	1	0.0939	1.8716	0.2136
a^2	4.7109	1	4.7109	93.8809	< 0.0001
b^2	1.3871	1	1.3871	27.6428	0.0012
c^2	0.8799	1	0.8799	17.5358	0.0041
Residual	0.3513	7	0.0502		
Lack of fit	0.0014	3	0.0005	0.0054	0.9994
Pure error	0.3498	4	0.0875		
R^2	0.9636				
Adj R^2	0.9169				

Table S3. Analysis of variance for regression model equation (20-hydroxyecdysone content).

Source	Sum of squares	df	Mean square	F value	p-value prob > F
Model	0.1354	9	0.0150	24.8548	0.0002
a	0.0045	1	0.0045	7.3541	0.0301
b	0.0027	1	0.0027	4.4102	0.0739

c	0.0040	1	0.0040	6.6164	0.0369
ab	0.0047	1	0.0047	7.8128	0.0267
ac	0.0006	1	0.0006	0.9476	0.3628
bc	0.0135	1	0.0135	22.3573	0.0021
a^2	0.0324	1	0.0324	53.5689	0.0002
b^2	0.0099	1	0.0099	16.3002	0.0049
c^2	0.0534	1	0.0534	88.1754	<0.0001
Residual	0.0042	7	0.0006		
Lack of fit	0.0026	3	0.0009	2.0255	0.2529
Pure error	0.0017	4	0.0004		
R^2	0.9697				
Adj R^2	0.9306				

Table S4. NMR data of compound **2** in CD_3OD.

Position	^{1}H (*J* value)	DEPT	^{13}C
2		C	157.0
3		C	133.5
4		C	178.0
5		C	161.3
6	6.17 *d* (1.9)	CH	98.3
7		C	164.3
8	6.37 *d* (1.9)	CH	93.2
9		C	156.9
10		C	104.3
1′		C	121.8
2′	7.71 *d* (2.1)	CH	115.9
3′		C	144.4
4′		C	148.3
5′	6.86 *d* (8.4)	CH	114.7
6′	7.62 *d* (8.4, 2.1)	CH	121.8
Galactose			
1″	5.40 *d* (7.9)	CH	100.3
2″	3.99 *dd* (7.9, 9.4)	CH	74.6
3″	3.79 (*m*)	CH	73.4
4″	3.55 d (3.5)	CH	68.3
5″	3.71 (*m*)	CH	73.3
6″a	3.42 (12.6, 6.8)	CH_2	65.5
6″b	3.70 (12.6, 4.7)		
Ramnose			
1″″	4.52 *d* (1.5)	CH	100.6
2″″	3.57 (3.4, 1.5)	CH	70.8
3″″	3.78 *d* (3.4)	CH	69.3
4″″	3.27 *t* (9.4)	CH	72.6
5″″	3.51 *dq* (9.4, 6.2)	CH	71.8
6″″	1.18 *d* (6.2)	CH_3	16.5
Apiose			
1′′′	5.46 *d* (1.2)	CH	109.5
2′′′	4.06 *d* (1.2)	CH	76.6
3′′′		C	79.4
4′′′ a	3.68 *d* (10)	CH_2	74.2
4′′′ b	4.04 *d* (10)		
5′′′	3.61 *s*	CH_2	64.8

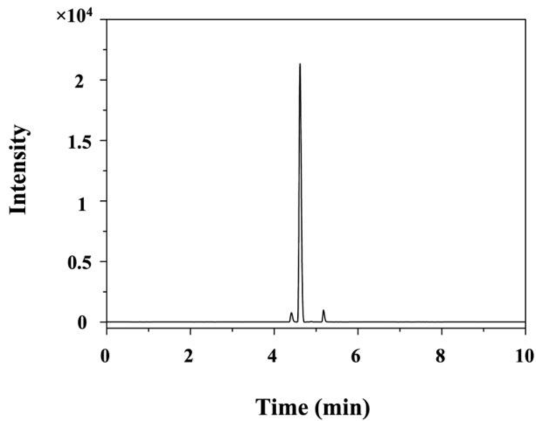

Figure S1. UPLC-QqQ-MS/MS spectrum of standard 20-hydroxecdysone.

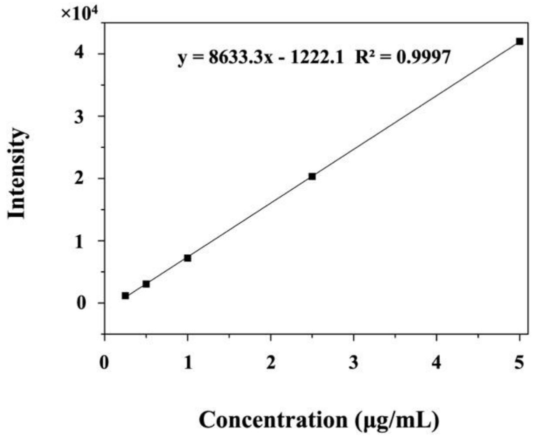

Figure S2. Standard curve of 20-hydroxecdysone.

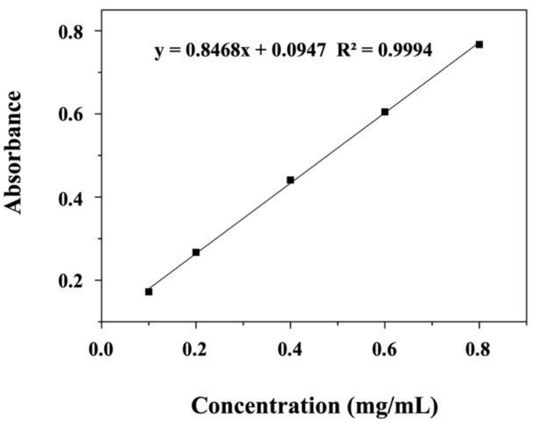

Figure S3. Standard curve of TF.

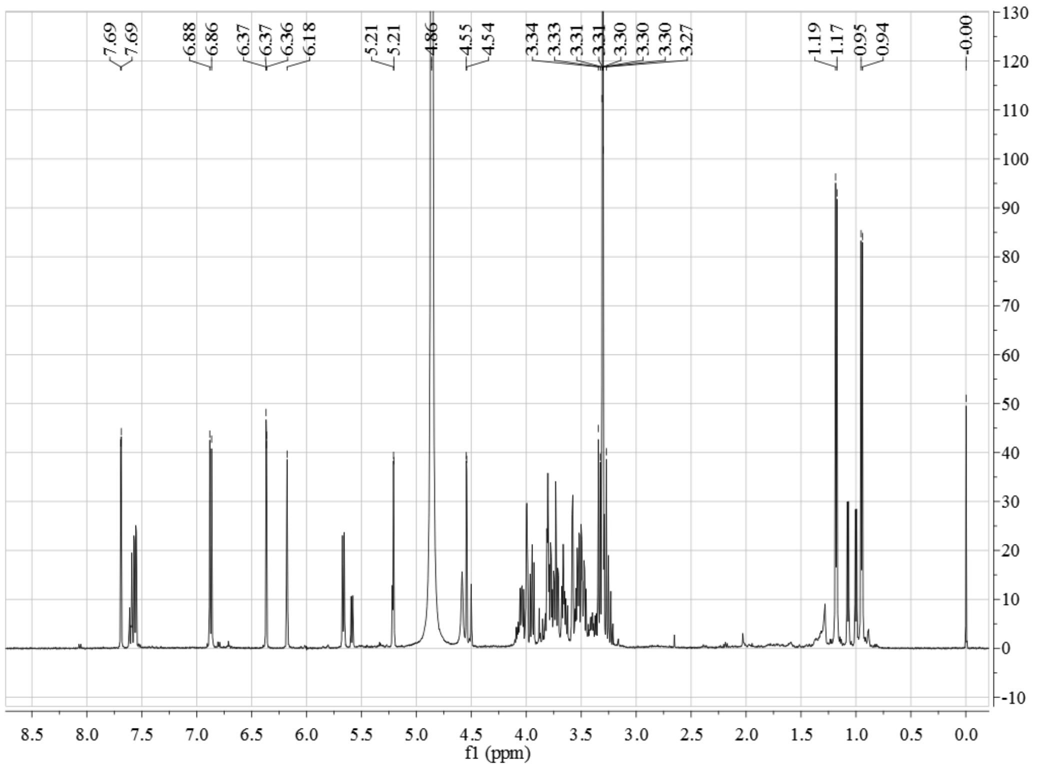

Figure S4. ^{1}H NMR of compound **1**.

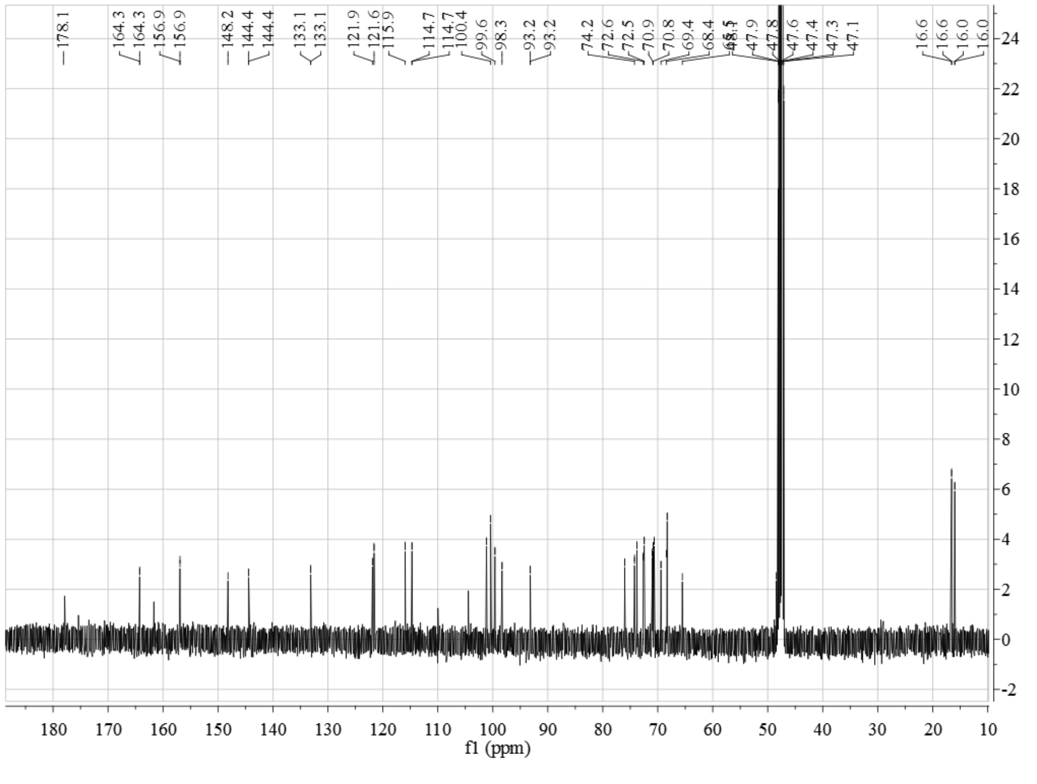

Figure S5. ^{13}C NMR of compound **1**.

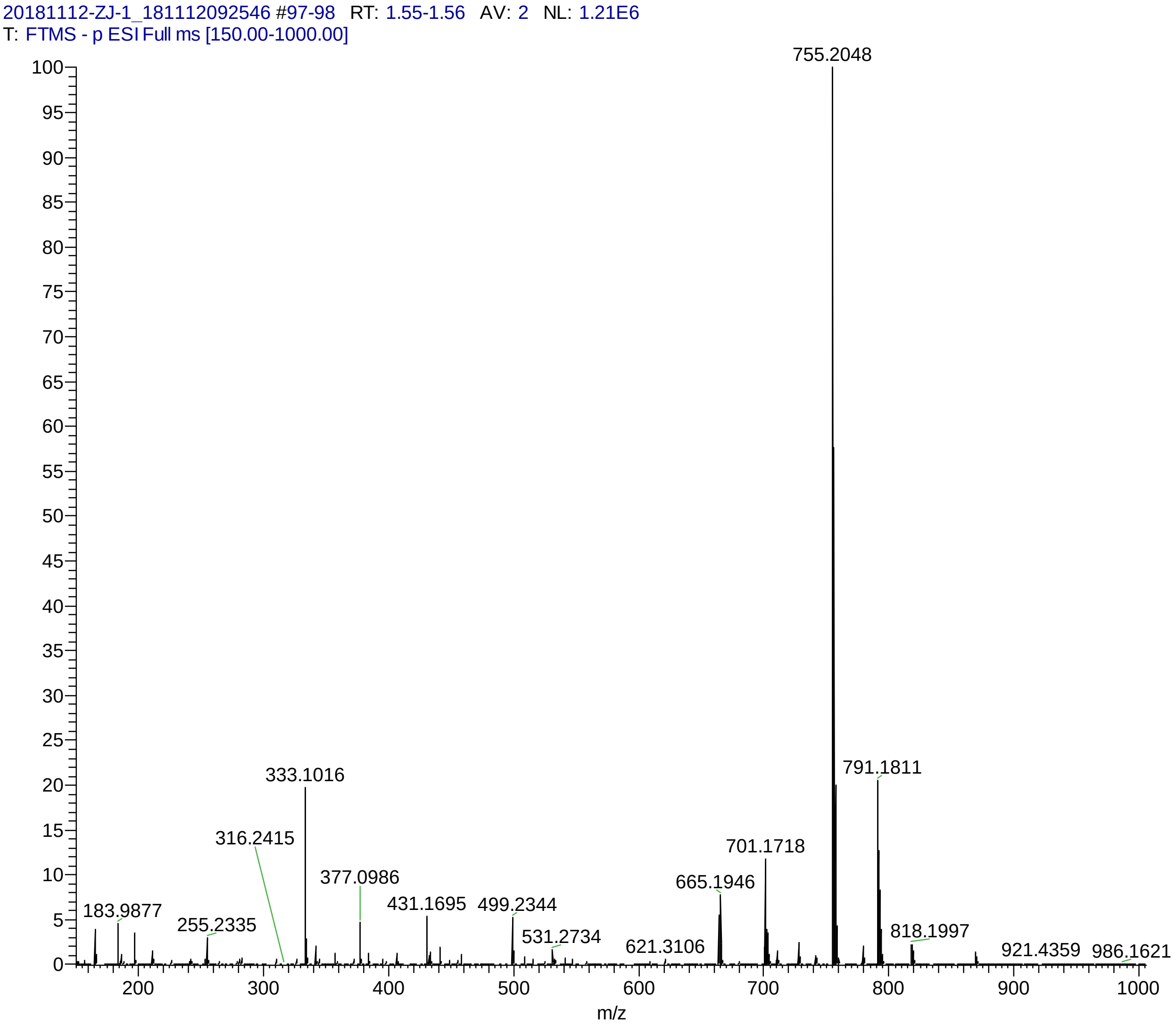

Figure S6. HRESI-MS of compound **1**.

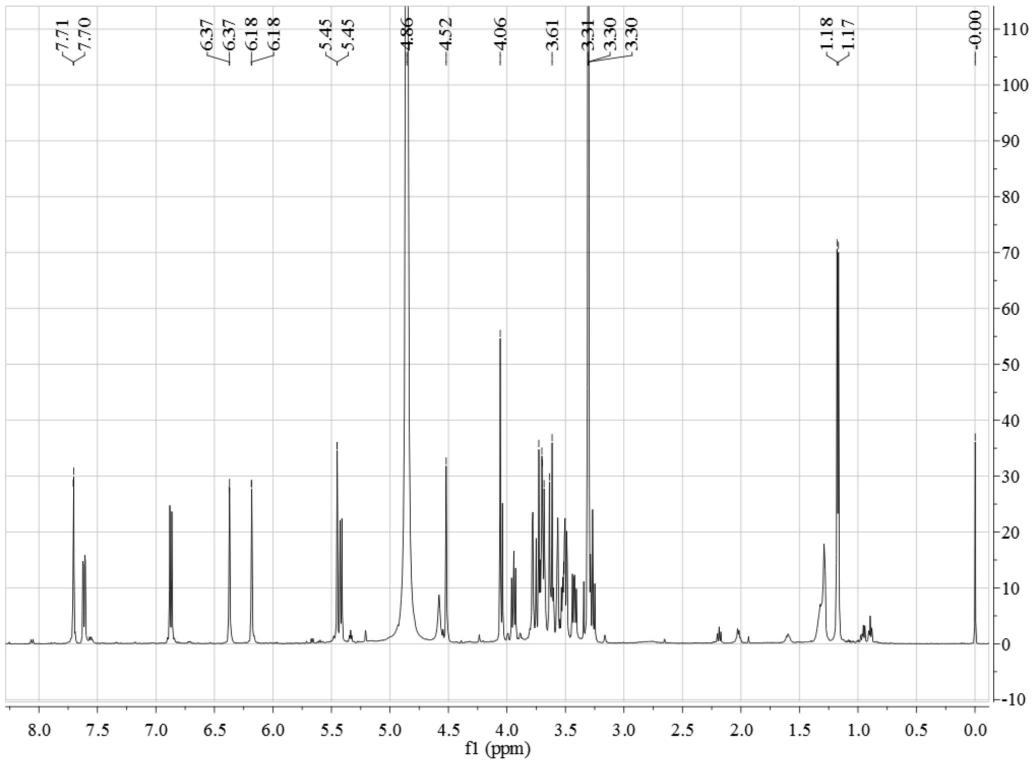

Figure S7. ^{1}H NMR of compound **2**.

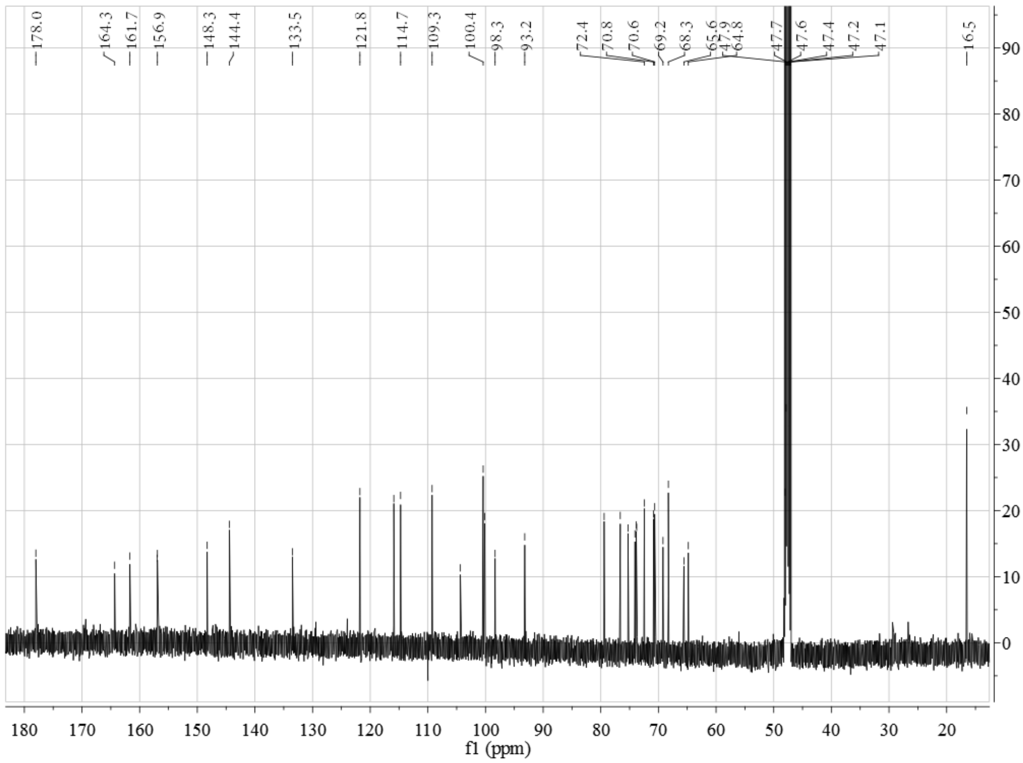

Figure S8. ^{13}C NMR of compound **2**.

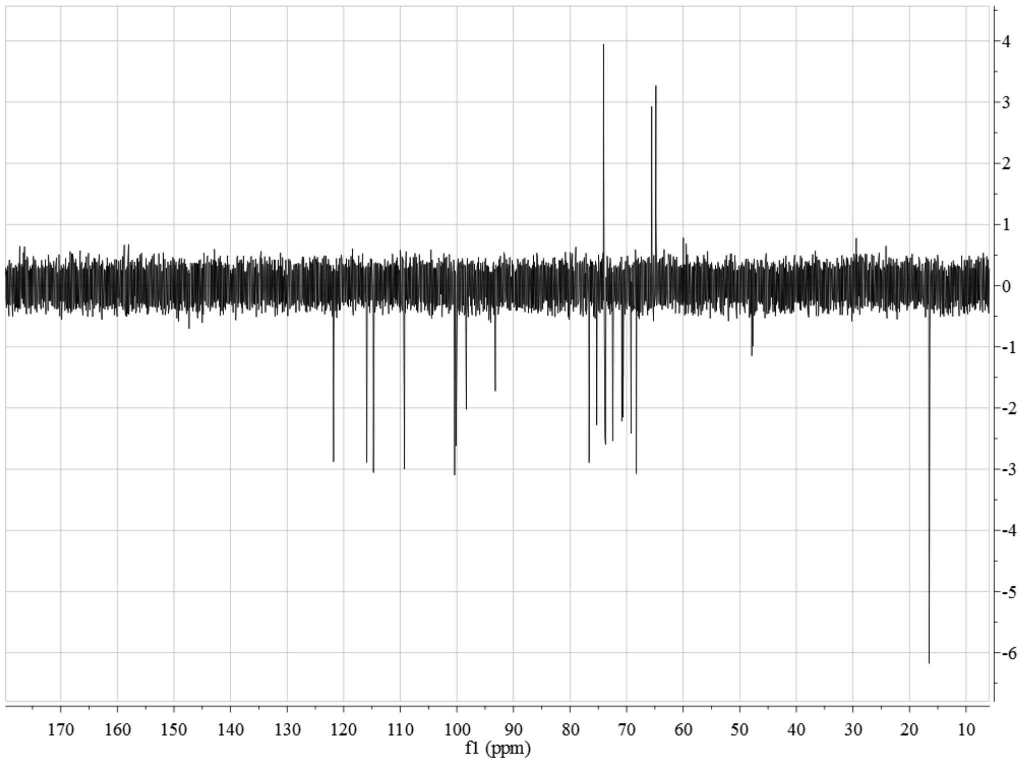

Figure S9. DEPT (135°) of compound **2**.

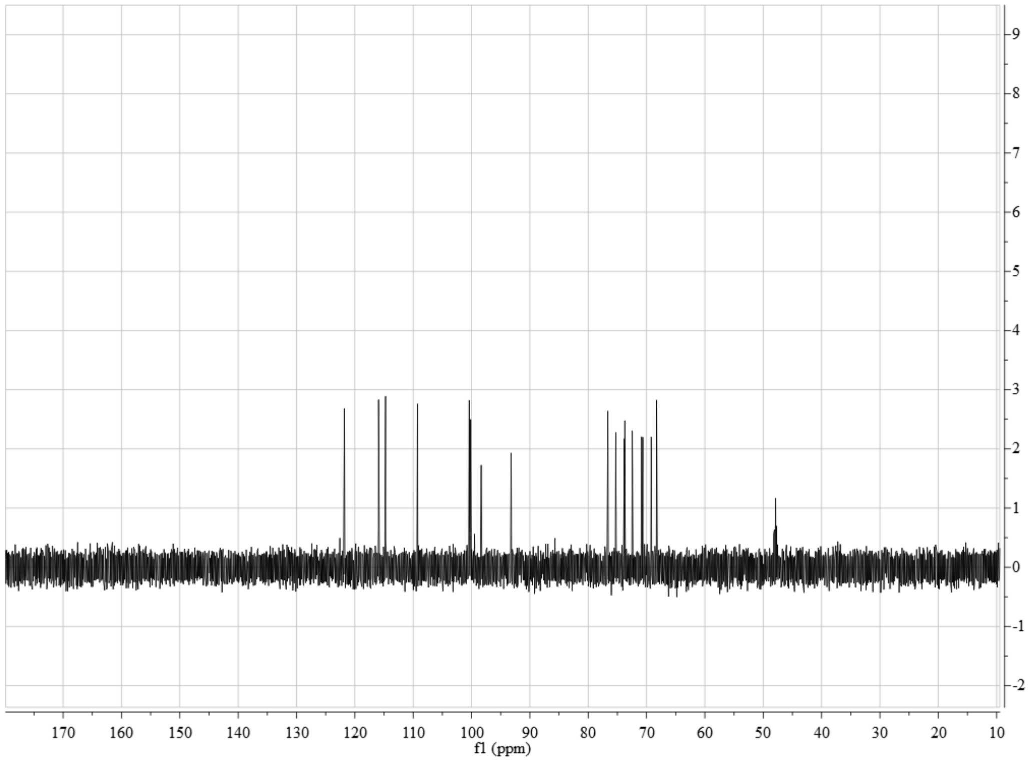

Figure S10. DEPT (90°) of compound **2**.

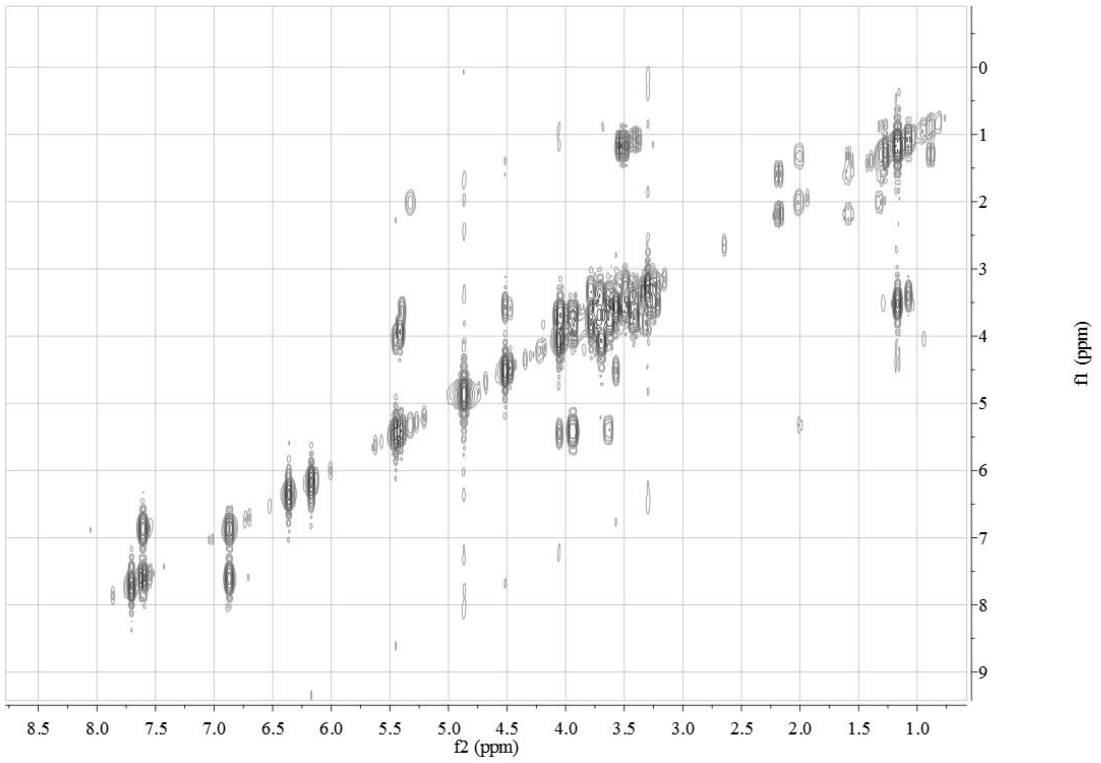

Figure S11. ^{1}H-^{1}H COSY of compound **2**.

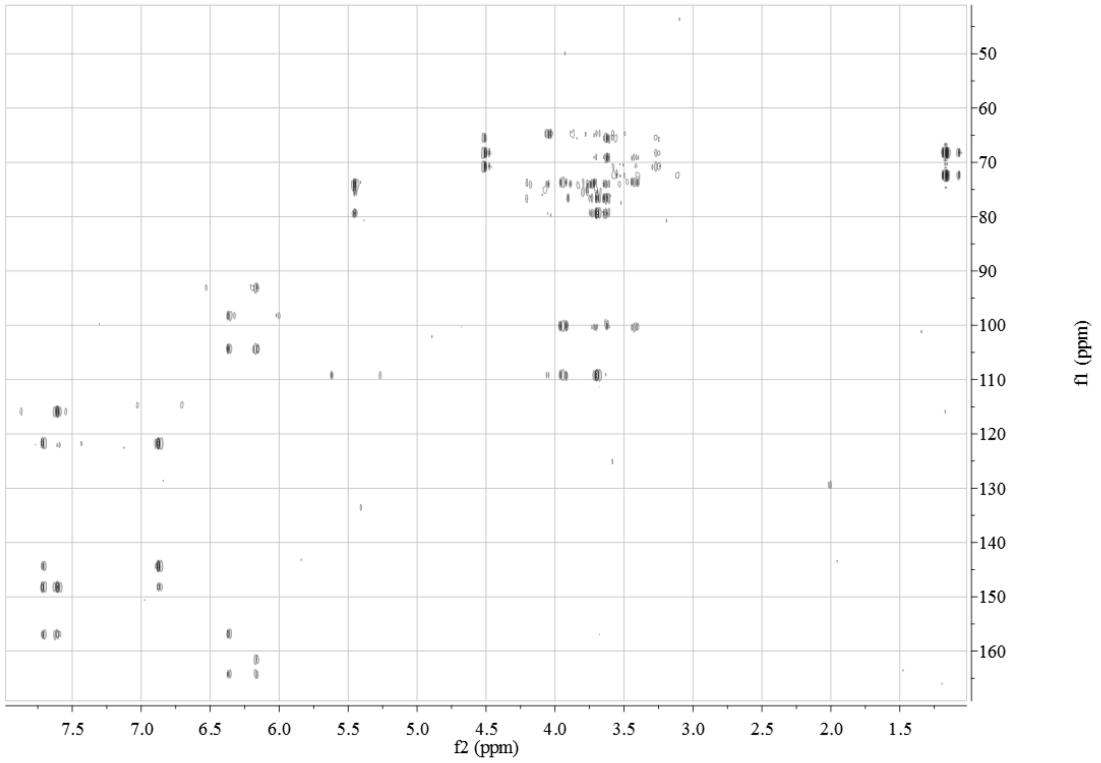

Figure S12. HMBC of compound **2**.

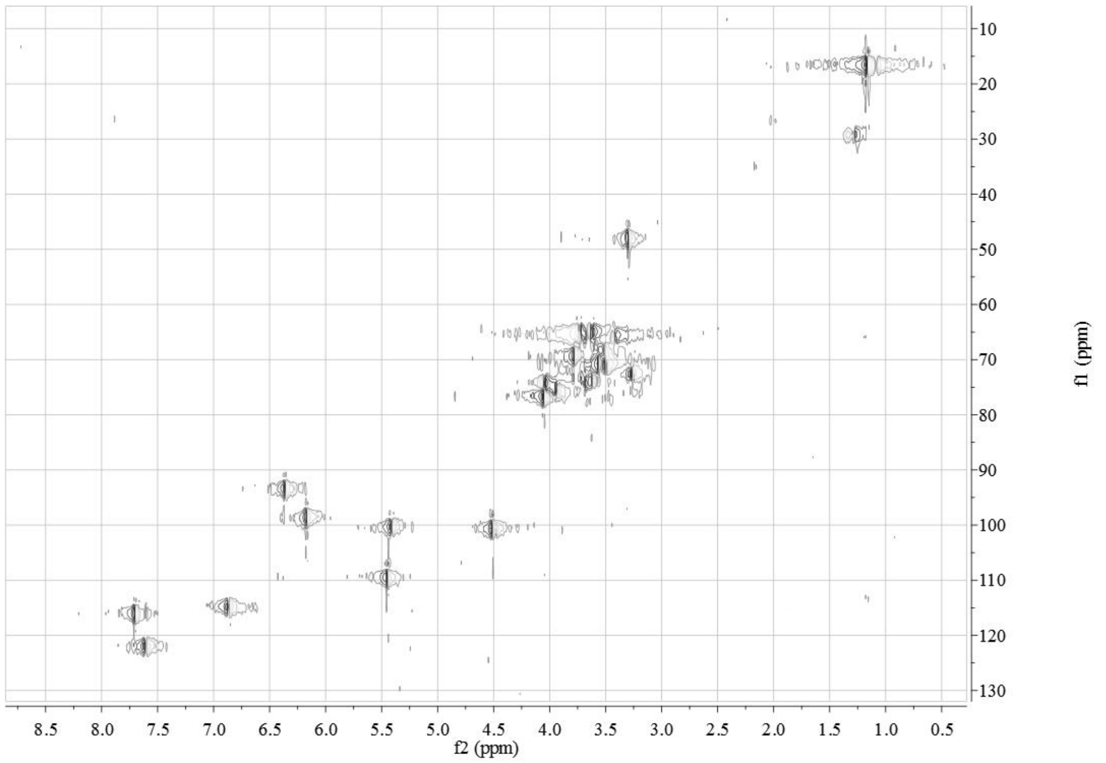

Figure S13. HSQC of compound **2**.

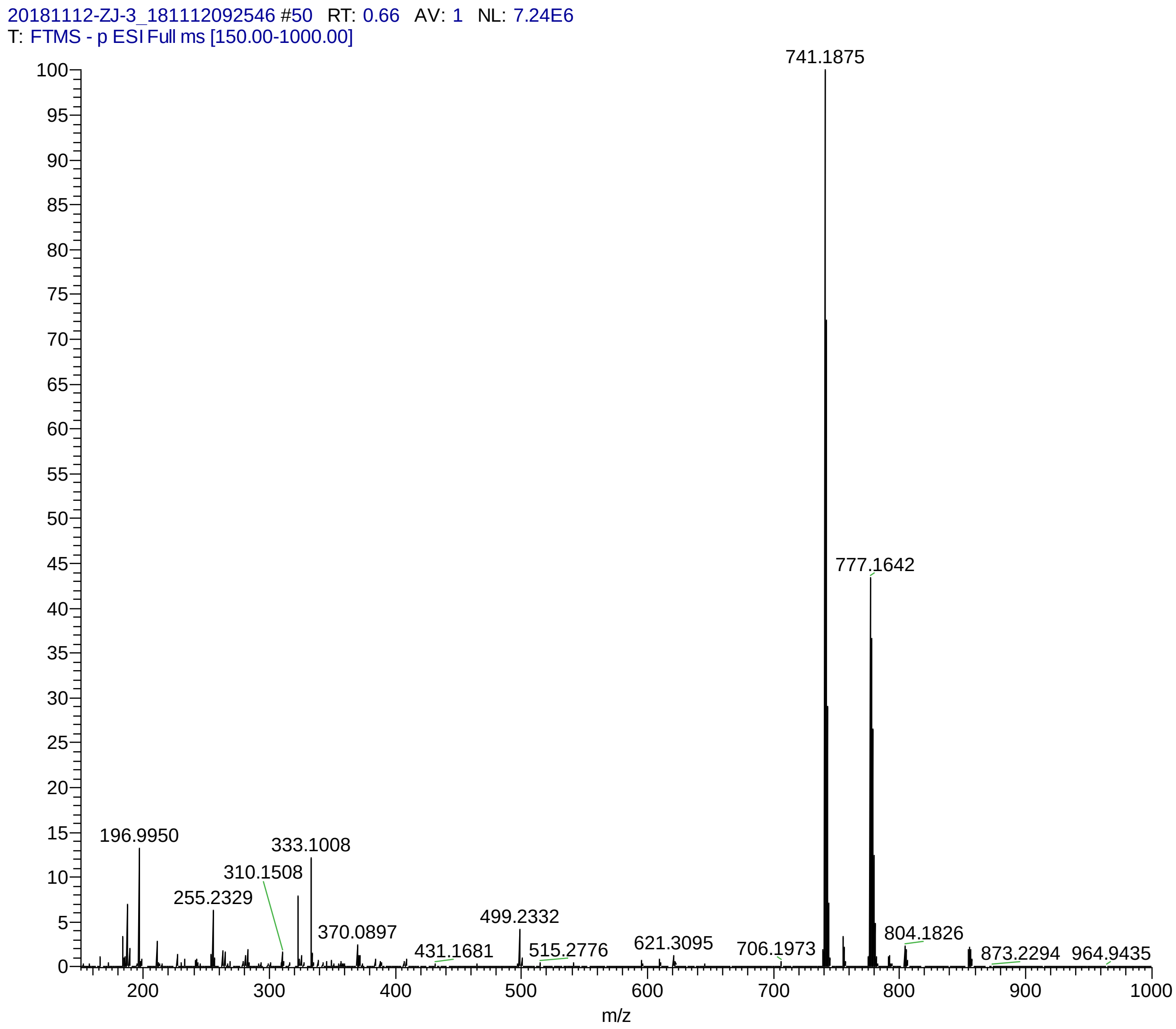

Figure S14. HRESI-MS of compound **2**.

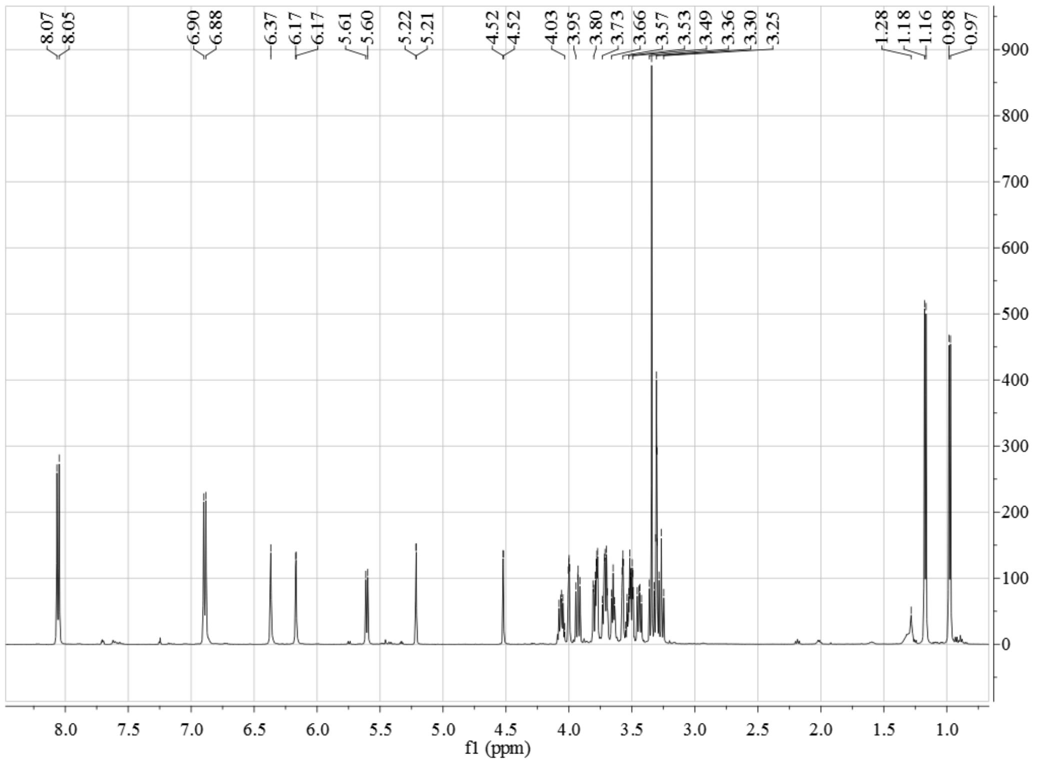

Figure S15. ^{1}H NMR of compound **3**.

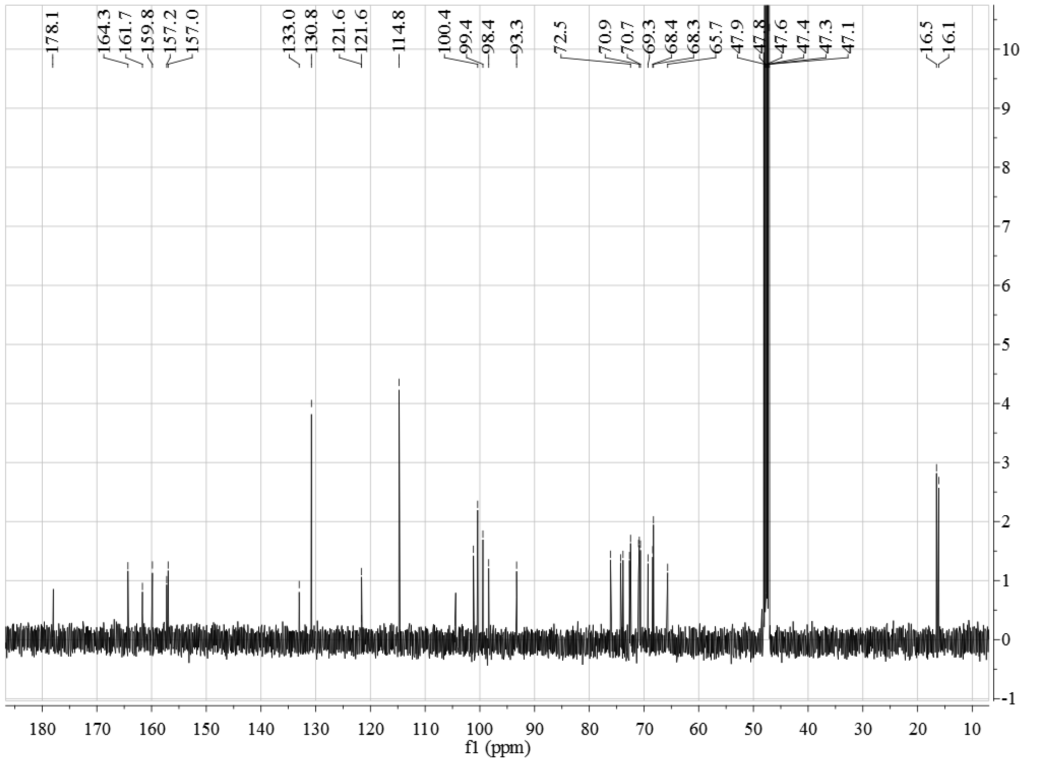

Figure S16. ^{13}C NMR of compound **3**.

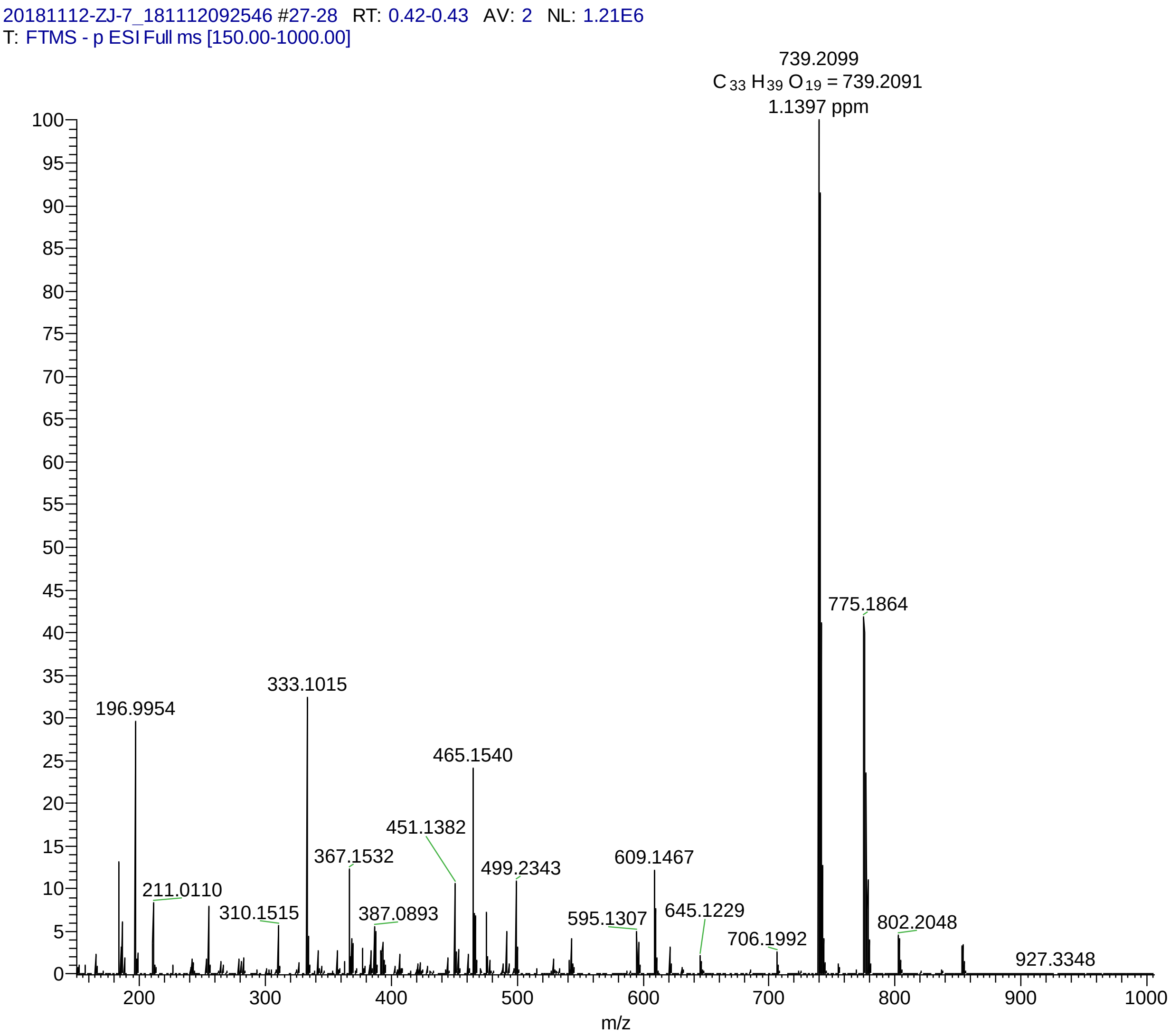

Figure S17. HRESI-MS of compound **3**.

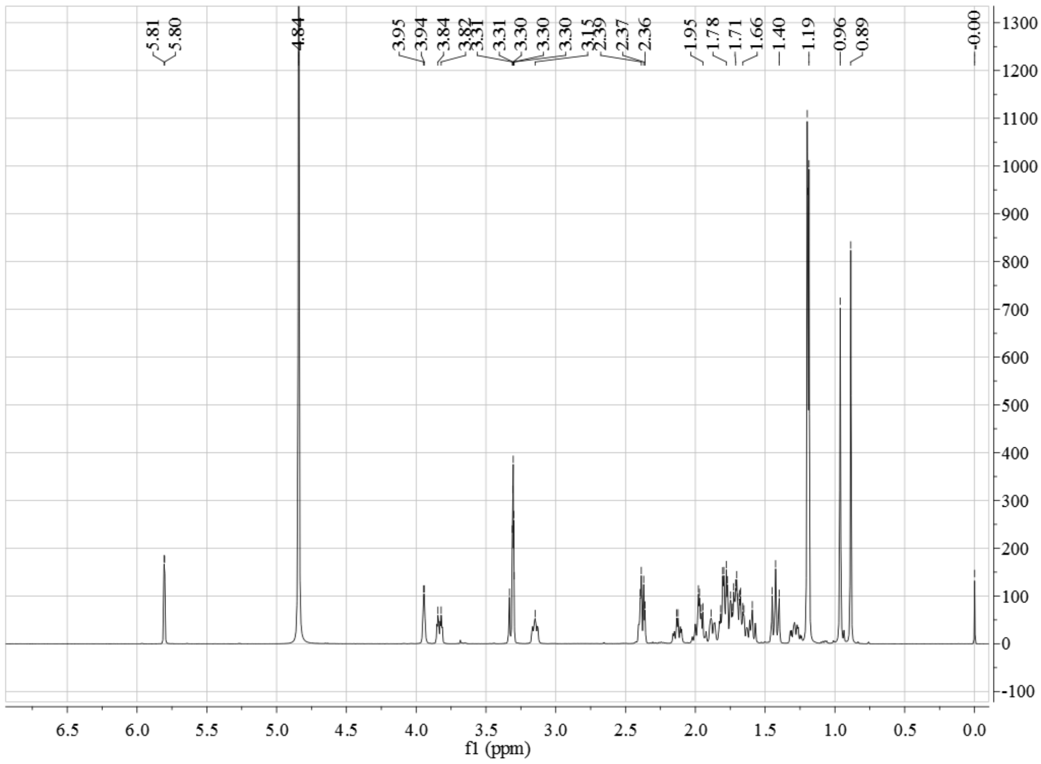

Figure S18. ^{1}H NMR of compound **4**.

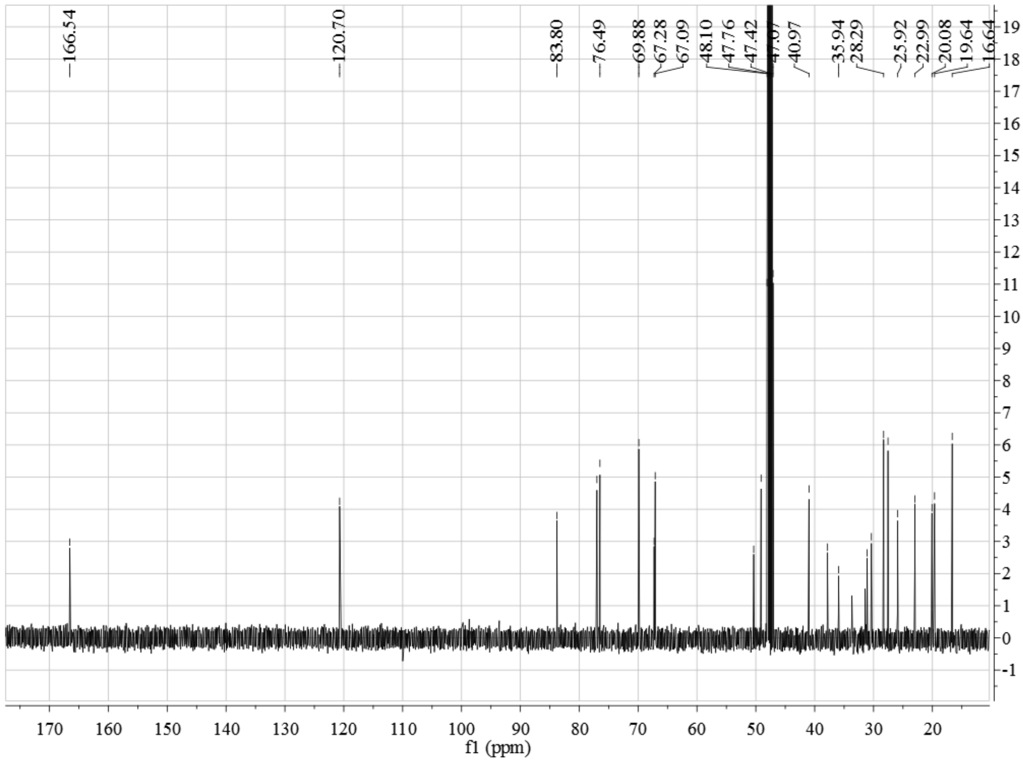

Figure S19. ^{13}C NMR of compound **4**.

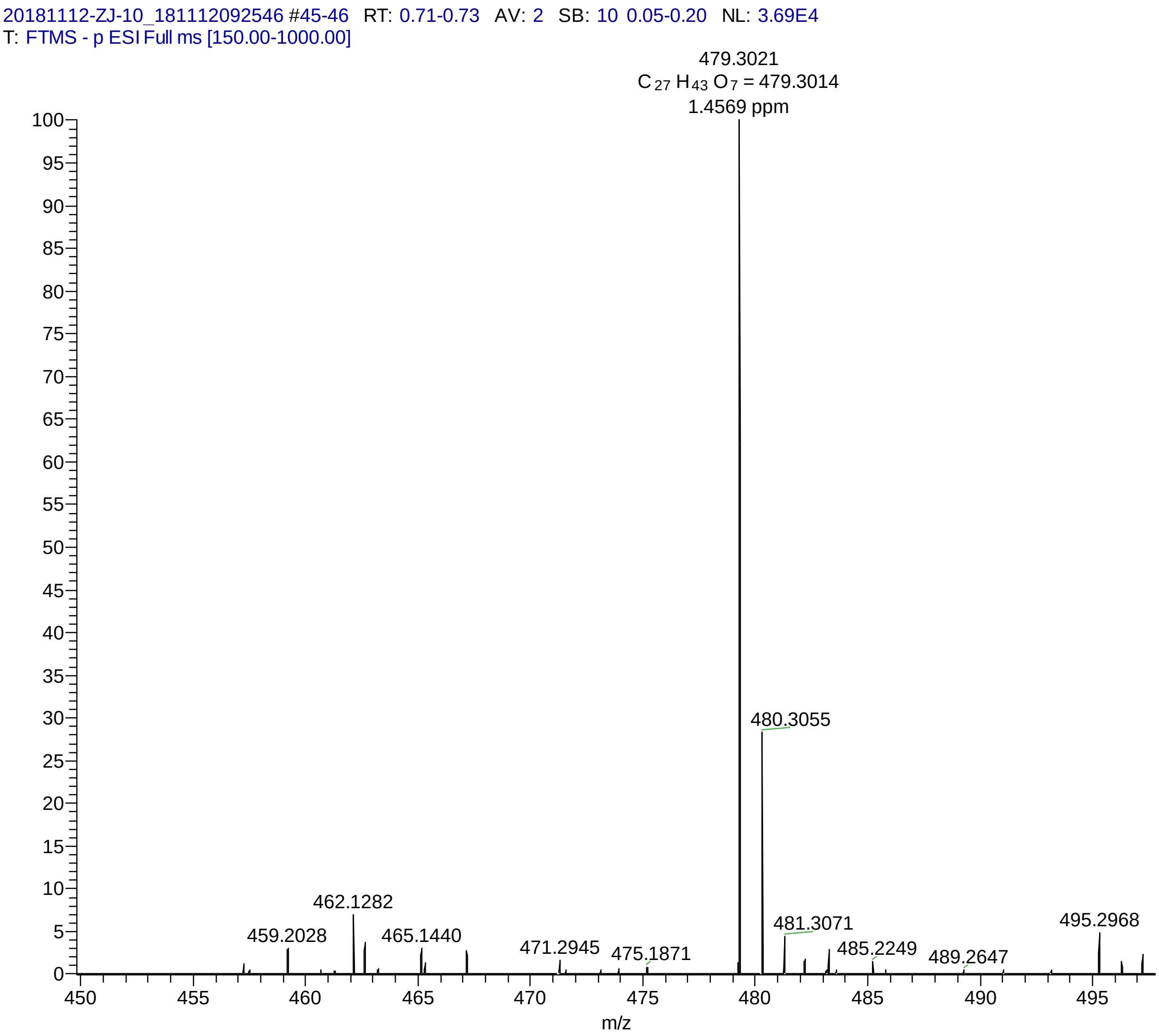

Figure S20. HRESI-MS of compound **4**.
